# The *C. elegans* D2-Like Dopamine Receptor DOP-3 Decreases Behavioral Sensitivity to the Olfactory Stimulus 1-Octanol

**DOI:** 10.1371/journal.pone.0009487

**Published:** 2010-03-02

**Authors:** Meredith J. Ezak, Denise M. Ferkey

**Affiliations:** Department of Biological Sciences, State University of New York at Buffalo, Buffalo, New York, United States of America; Harvard University, United States of America

## Abstract

We previously found that dopamine signaling modulates the sensitivity of wild-type *C. elegans* to the aversive odorant 1-octanol. *C. elegans* lacking the CAT-2 tyrosine hydroxylase enzyme, which is required for dopamine biosynthesis, are hypersensitive in their behavioral avoidance of dilute concentrations of octanol. Dopamine can also modulate the context-dependent response of *C. elegans* lacking RGS-3 function, a negative regulator of Gα signaling. *rgs-3* mutant animals are defective in their avoidance of 100% octanol when they are assayed in the absence of food (*E. coli* bacterial lawn), but their response is restored when they are assayed in the presence of food or exogenous dopamine. However, it is not known which receptor might be mediating dopamine's effects on octanol avoidance. Herein we describe a role for the *C. elegans* D2-like receptor DOP-3 in the regulation of olfactory sensitivity. We show that DOP-3 is required for the ability of food and exogenous dopamine to rescue the octanol avoidance defect of *rgs-3* mutant animals. In addition, otherwise wild-type animals lacking DOP-3 function are hypersensitive to dilute octanol, reminiscent of *cat-2* mutants. Furthermore, we demonstrate that DOP-3 function in the ASH sensory neurons is sufficient to rescue the hypersensitivity of *dop-3* mutant animals, while *dop-3* RNAi knockdown in ASH results in octanol hypersensitivity. Taken together, our data suggest that dopaminergic signaling through DOP-3 normally acts to dampen ASH signaling and behavioral sensitivity to octanol.

## Introduction

With the possible exception of insects, olfaction is mediated by G protein-coupled signal transduction pathways across species [1]–[6]. Odorant ligands bind to 7-transmembrane G protein-coupled receptors (GPCRs) expressed in olfactory sensory neurons. This binding induces a conformational change in the receptor that activates the associated heterotrimeric G proteins. Gα exchanges GDP for GTP and, once dissociated, the Gα-GTP and Gβγ subunits can activate distinct downstream targets and second messenger generating systems within the cell.

The *C. elegans* genome encodes >500 predicted functional chemosensory GPCRs and, as in other organisms, olfactory signaling in *C. elegans* is mediated by G protein-coupled signaling cascades [1]–[3]. G protein-coupled pathways in the AWA and AWC chemosensory neurons mediate chemotaxis towards attractive odorants that likely signal the presence of a food source, while the ASH, AWB and ADL neurons detect aversive odorants that might indicate an unfavorable or harmful environment [1]. The well-characterized polymodal ASH sensory neurons actually detect a wide range of aversive stimuli, including volatile odorants (e.g. octanol), soluble chemicals (e.g. quinine), high osmolarity and the mechanical stimulus of light touch to the nose [7]–[11]. Animals exhibit an avoidance response by rapidly initiating backwards locomotion upon detection of any of these stimuli.

To allow for appropriate cellular and organismal responses to these environmental stimuli, the level and duration of signaling through GPCRs must be precisely controlled. In the ASH neurons, this is accomplished in part by GRK (G protein-coupled receptor kinase) and RGS (regulator of G protein signaling) proteins [12], [13]. Generally, GRKs phosphorylate activated GPCRs to downregulate receptor signaling [14]–[16], while RGS GTPase-activating proteins bind to Gα-GTP and accelerate the rate of GTP hydrolysis to downregulate signaling at the level of G proteins [17]. In addition, biogenic amines (dopamine, serotonin, tyramine and octopamine) alter the sensitivity of *C. elegans* to sensory stimuli that are detected by ASH [10], [13], [18]–[20]. However, in some cases the receptors for these biogenic amines function in cells besides ASH to modulate ASH-mediated behavioral responses [18], [20].

Dopamine (DA) and serotonin (5-HT) are believed to signal the presence of food for *C. elegans* [21]–[28], and the presence of food or exogenous 5-HT enhances behavioral responses to the aversive stimuli of nose touch and diluted octanol [18]–[20]. Exogenous tyramine (TA) or octopamine (OA) can counter this effect and block the food or 5-HT-dependent increase in dilute octanol sensitivity [18]. Loss of the *cat-2 tyrosine hydroxylase* gene, which encodes an enzyme required specifically for dopamine (DA) biosynthesis [29], renders animals hypersensitive to dilute concentrations of the aversive odorant octanol, suggesting that DA normally dampens chemosensory signaling in wild-type animals as well [13], [18]. Combined, these results suggest that endogenous 5-HT may act to enhance sensory signaling and behavioral responsiveness to aversive stimuli when animals are in a food rich environment, while TA, OA and DA may dampen behavioral responses.

DA also affects the ASH-mediated responses of *rgs-3* mutant animals [13]. *rgs-3* encodes an RGS protein that functions in some *C. elegans* sensory neurons, including ASH [13], and *rgs-3* mutants are defective in their responses to strong chemosensory and mechanosensory stimuli in the absence of food (*E. coli* bacterial lawn). *C. elegans* lacking RGS-3 function seem to have behavioral defects because increased signaling in the ASH sensory neurons ultimately leads to decreased glutamatergic signaling at the sensory/interneuron synapse [13]. Accordingly, addition of exogenous serotonin, which enhances signaling and further increases Ca^2+^ transients in the ASH neurons [10], exacerbates the *rgs-3* behavioral defects [13]. However, the responses of *rgs-3* mutants are significantly improved when assayed in the presence of either food or DA [13]. These results suggest that food restores *rgs-3* behavioral responses by activating an inhibitory dopaminergic pathway that dampens the increased signaling levels in ASH in the absence of RGS-3 function. Furthermore, as food rescues the *rgs-3* behavioral deficit, yet signals the release of both DA and 5-HT, this suggests that endogenous DA signaling may override the effect of 5-HT on the ASH chemosensory signaling circuit. This is consistent with the observation that exogenous DA blocks the 5-HT-dependent increases in the octanol sensitivity of wild-type animals [18]. It remains unclear, however, which receptors are mediating DA's effects in wild-type or *rgs-3* mutants.

Dopaminergic signaling is highly conserved across species. Dopamine receptors are generally grouped into two classes: D1-like receptors signal through G_s_α/G_olf_α to increase adenylate cyclase activity and cAMP levels in target cells, while stimulation of D2-like receptors couples to G_i_α/G_o_α subunits and leads to an inhibition of adenylate cyclase and a decrease in cAMP levels [30]. In *C. elegans*, as in vertebrates, dopamine can activate G protein-coupled signaling pathways, and candidate receptors have not only been shown to bind the neurotransmitter, but also to neurotransmitter agonists and antagonists [31], [32]. In addition to octanol sensitivity [13], [18], DA modulates a wide range of *C. elegans* behaviors, including food sensing, area restricted search, locomotion, egg-laying, defecation, state-dependent olfactory adaptation and habituation to non-localized mechanical stimulation (tap) [31], [32]. However, the mechanisms underlying DA's role in these behaviors are not as well understood.

The *C. elegans* genome encodes one D1-like DA receptor (DOP-1), two D2-like receptors (DOP-2 and DOP-3) and one invertebrate specific D1-like receptor (DOP-4) [25], [31]–[36]. Similar to loss of CAT-2 [13], [18], simultaneous loss of three *C. elegans* DA receptors (DOP-1, DOP-2 and DOP-3) resulted in hypersensitivity to dilute octanol [18]. However, the effect of individual DA receptors on octanol response was not determined. Because different receptors can couple to unique downstream pathways, we sought to determine whether an individual receptor is responsible for dopaminergic modulation of octanol avoidance, or whether multiple pathways might exert an additive effect on behavior. We show here that only DOP-3 is required for the ability of either food or exogenous DA to rescue the octanol response defect of *rgs-3* animals, suggesting that in well fed animals endogenous DA signals through DOP-3 to modulate octanol behavioral sensitivity. We also show that loss of DOP-3 function in otherwise wild-type animals results in hypersensitivity to dilute octanol, suggesting that endogenous DA normally dampens octanol sensitivity via DOP-3. While DOP-3 transgene reporter expression was not observed in ASH, DOP-3 expression in ASH is sufficient to rescue the octanol hypersensitivity of *dop-3* mutant animals, while loss of DOP-3 function in ASH leads to octanol hypersensitivity. Combined, we have uncovered a role for the dopamine receptor DOP-3 in the modulation of octanol sensitivity in *C. elegans*.

## Results

### DOP-3 Is Required for *rgs-3* Avoidance of 100% Octanol in the Presence of Food

**Figure 1:**
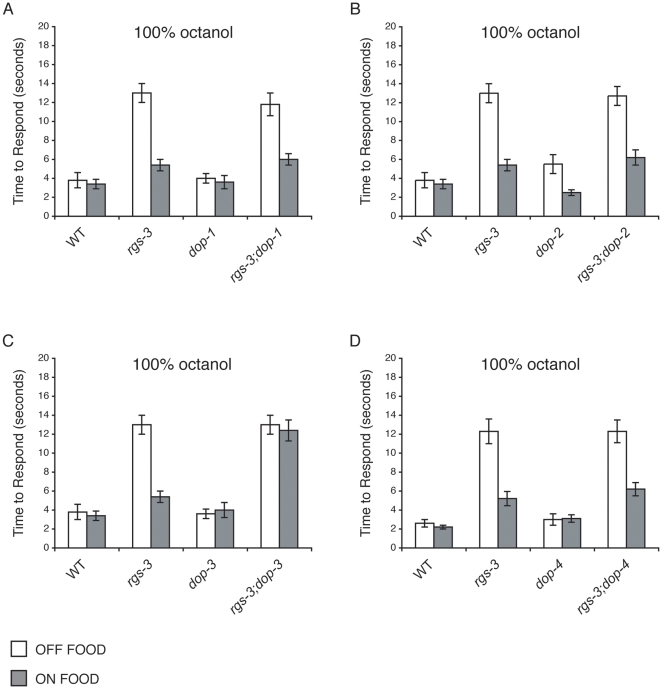
DOP-3 is required for the “on food” rescue of *rgs-3* octanol avoidance. Food (OP50 *E. coli*) restores the avoidance response of *rgs-3* mutant animals to 100% octanol. Loss-of-function mutations in (A) *dop-1*, (B) *dop-2* and (D) *dop-4* had no effect on the “on food” rescue of *rgs-3* octanol avoidance (p>0.2 for each when compared to the *rgs-3* on food response). (C) Loss of DOP-3 function blocked the “on food” rescue of *rgs-3* octanol avoidance (p≤0.0001 when compared to *rgs-3* on food). Alleles used: *rgs-3(vs19)*, *dop-1(vs101)*, *dop-2(vs105)*, *dop-3(vs106)* and *dop-4(tm1392)*. WT  =  the N2 wild-type strain. The time to respond is shown. n≥32. Error bars represent the standard error of the mean (SEM).

Animals lacking RGS-3 function are defective in their avoidance of 100% octanol when assayed in the absence of food (*E. coli* bacteria). However, *rgs-3* animals respond significantly better when they are assayed in the presence of food or exogenous dopamine (DA) [13]. The *C. elegans* genome encodes 4 putative DA GPCRs: DOP-1 (D1-like), DOP-2 (D2-like), DOP-3 (D2-like) and DOP-4 (invertebrate specific D1-like). To determine which DA receptor(s) might contribute to the food and DA rescue of *rgs-3* octanol avoidance, *rgs-3* animals lacking each of the DA receptors were assayed for octanol avoidance in the absence and presence of the bacterial food lawn. Each of the double mutants displayed defective octanol avoidance in the absence of food, taking ∼12 seconds to respond, similar to *rgs-3* animals (Figure 1). Loss of DOP-1, DOP-2 or DOP-4 had no effect on the food rescue of *rgs-3* octanol avoidance, while *rgs-3;dop-3* animals remained defective for octanol avoidance when assayed on food (Figure 1). This indicates that DOP-3 is required for *rgs-3* animals to avoid 100% octanol in the presence of food.

### DOP-1 Does Not Antagonize DOP-3 in Octanol Avoidance

**Figure 2:**
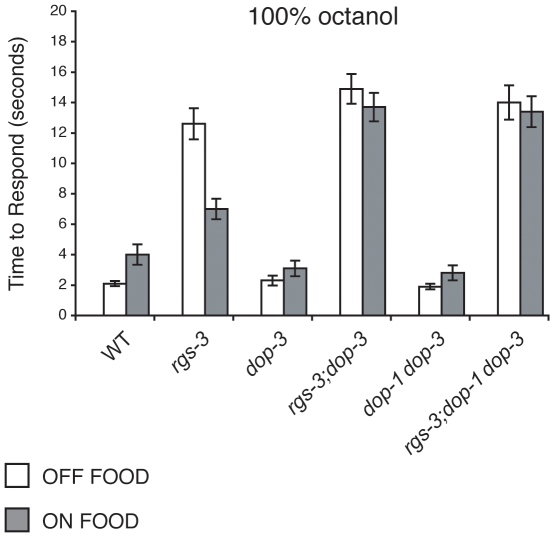
DOP-1 does not antagonize DOP-3 in octanol avoidance. Previous studies showed that DOP-1 can antagonize DOP-3 in cholinergic motor neurons [25]. However, loss of DOP-1 function had no effect on the avoidance of 100% octanol when examined in combination with loss of DOP-3 function. The “on food” response of *rgs-3;dop-3* animals was indistinguishable from that of *rgs-3;dop-1dop-3* (p>0.5). Alleles used: *rgs-3(vs19)*, *dop-1(vs100)* and *dop-3(vs106)*. WT  =  the N2 wild-type strain. The time to respond is shown. n≥40. Error bars represent the standard error of the mean (SEM).

Previous work showed that DOP-1 antagonizes DOP-3 in the cholinergic motor neurons to regulate locomotion behaviors such as the basal slowing response when animals encounter a food source and paralysis caused by the addition of high concentrations of exogenous DA [25]. Importantly, while *dop-1* mutant animals did not show a defect and responded similarly to wild-type animals, loss of DOP-1 countered loss of DOP-3; a role for DOP-1 was only revealed when examined in combination with loss of DOP-3 [25]. To determine whether DOP-1 might also antagonize DOP-3 in the regulation of octanol avoidance, *rgs-3;dop-3* animals were compared to *rgs-3;dop-1dop-3* animals for avoidance of 100% octanol off and on food. Both remained defective for octanol avoidance when assayed on food (Figure 2), suggesting that DOP-1 does not contribute to the regulation of octanol avoidance.

### DOP-3 Is Required for *rgs-3* Avoidance of 100% Octanol in the Presence of Exogenous Dopamine

**Figure 3:**
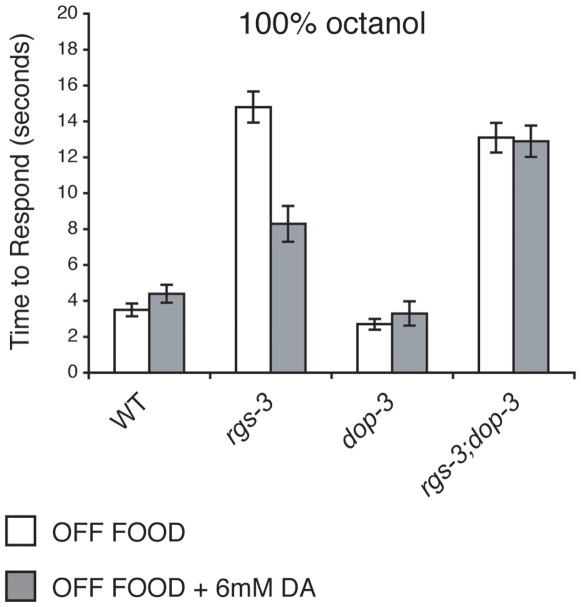
DOP-3 is required for the dopamine rescue of *rgs-3* octanol avoidance. Exogenous dopamine (DA) restores the avoidance response of *rgs-3* mutant animals to 100% octanol (p<0.0001 when comparing *rgs-3* +/− DA). Loss of DOP-3 function blocks the DA rescue of the *rgs-3* response to octanol (p>0.5 when comparing *rgs-3;dop-3* +/− DA). Alleles used: *rgs-3(vs19)* and *dop-3(vs106)*. WT  =  the N2 wild-type strain. The time to respond is shown. n≥40. Error bars represent the standard error of the mean (SEM).

When animals are assayed in the absence of food, exogenous DA is sufficient to partially restore *rgs-3* animals' response to 100% octanol [13]. As loss of DOP-3 blocked the food rescue of *rgs-3* octanol avoidance (Figure 1), we assessed whether DOP-3 was required for exogenous DA to rescue *rgs-3* octanol avoidance. While *rgs-3* animals responded significantly better to 100% octanol in the presence of 6mM DA, *rgs-3;dop-3* animals remained defective in their response even in the presence of exogenous DA (Figure 3). Taken together, these results indicate that DOP-3 is required for both food and exogenous DA to restore octanol avoidance to *rgs-3* mutant animals.

### Animals Lacking DOP-3 Are Hypersensitive to Dilute Octanol

**Figure 4:**
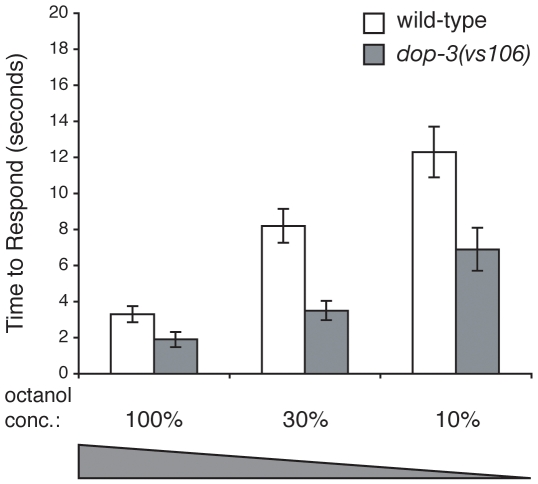
Loss of DOP-3 function results in enhanced sensitivity to dilute octanol. Loss of dopamine receptor DOP-3 function renders animals hypersensitive to dilute octanol. *dop-3* animals respond better than wild-type animals to dilute octanol (p<0.0001 for 30% octanol and p<0.01 for 10% octanol). Allele used: *dop-3(vs106)*. WT  =  the N2 wild-type strain. The time to respond is shown. n≥40. Error bars represent the standard error of the mean (SEM). Conc.  =  concentration.

*C. elegans cat-2* encodes a tyrosine hydroxylase required specifically for DA biosynthesis [29]. Although *cat-2* mutant animals do not completely lack endogenous DA (they still synthesize ∼40% of wild-type *C. elegans* DA levels), this is similar to what is seen in tyrosine hydroxylase-deficient mice [35], [37]. *cat-2* mutant animals are hypersensitive and respond better than wild-type animals to dilute concentrations of octanol [13], [18]. In addition, animals lacking three DA receptors (*dop-2;dop-1dop-3* triple mutants) are hypersensitive to dilute octanol [18]. The ability of DOP-3 to selectively modulate the octanol avoidance responses of *rgs-3* animals suggests that endogenous DA may signal through DOP-3 to regulate octanol sensitivity in wild-type animals. *dop-3* single mutants were assayed off food for avoidance of dilute (30% and 10%) octanol. At both concentrations, *dop-3* mutant animals responded better than wild-type animals (Figure 4). The enhanced sensitivity of *dop-3* animals to dilute octanol suggests that signaling through DOP-3 normally acts to dampen octanol responses.

### Animals Lacking DOP-3 Are Not Hypersensitive to Dilute Attractive Odorants

**Figure 5:**
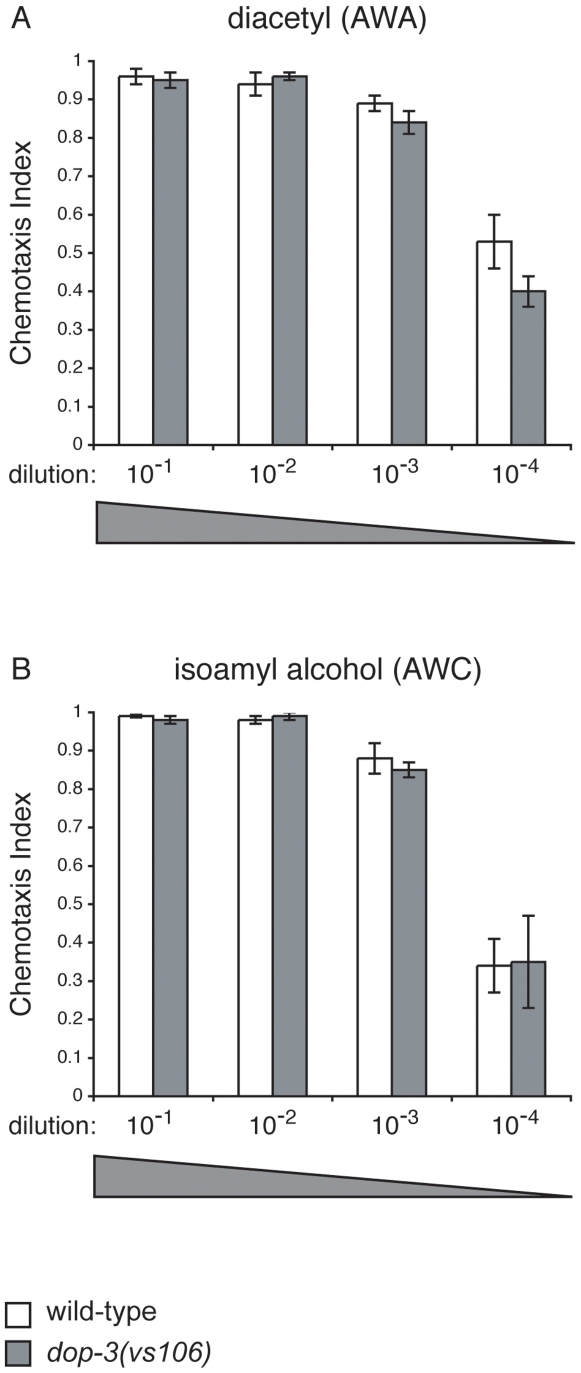
Loss of DOP-3 function does not result in hypersensitivity to attractive odorants. The AWA sensory neurons detect the attractive odorant diacetyl, while the AWC neurons detect the attractive odorant isoamyl alcohol [38]. Loss of dopamine receptor DOP-3 function did not lead to enhanced chemotactic responses to dilute concentrations of (A) diacetyl or (B) isoamyl alcohol (p>0.1 for all concentrations tested of both odorants). Chemotaxis index  =  (number of animals at odorant–number of animals at control) ÷ total number of animals on the assay plate. Each bar represents the average of ≥4 assays with 50–150 animals per trial. Allele used: *dop-3(vs106)*. WT  =  the N2 wild-type strain. Error bars represent the standard error of the mean (SEM).

Octanol is an aversive odorant detected by the ASH, AWB and ADL sensory neurons [9], [19]. The AWA and AWC olfactory neurons detect odorants that *C. elegans* are attracted to and chemotax towards [38]. To determine whether DOP-3 regulates olfactory responses generally, or is specific to ASH-mediated avoidance of octanol, *dop-3* animals were compared to wild-type animals for chemotaxis towards diacetyl (AWA) and isoamyl alcohol (AWC). A range of concentrations was tested for each odorant. In all cases, *dop-3* animals were indistinguishable from wild-type animals (Figure 5).

### DOP-3 Expression Is Not Seen in the Octanol-Detecting Neurons ASH, AWB and ADL

**Figure 6:**
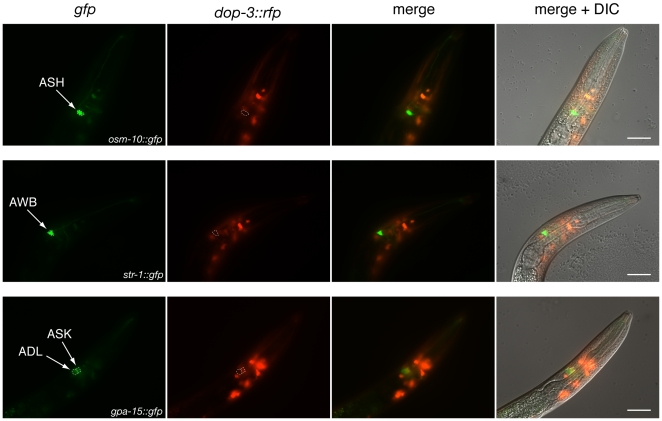
DOP-3::RFP expression is not seen in the sensory neurons that detect octanol. The ASH sensory neurons are the primary sensors of 100% octanol when animals are assayed on food, and only ASH is used to detect diluted octanol both on and off food [19]. The integrated transgene *vsIs33* encodes 13,035 base pairs of *dop-3* genomic DNA, including 9,955 base pairs of upstream promoter sequence and extending into the fourth coding exon [25]. This sequence is fused to the mRFP1 red fluorescent protein [25], [75]. DOP-3::RFP expression was not observed in ASH (marked by *osm-10::gfp*), or the other octanol-detecting neurons, AWB (marked by *str-1::gfp*) or ADL (marked by *gpa-15::gfp*). Weak expression was often observed in ASK (marked by *gpa-15::gfp*). Scale bar  = 20 µm.

*C. elegans* utilize different combinations of sensory neurons to detect octanol, depending on the feeding status of the animal and the octanol concentration. While the ASH, AWB and ADL neurons all contribute to the detection of 100% octanol off food, ASH is the primary 100% octanol-sensing neuron on food [19], [38]. Conversely, only ASH detects diluted octanol, independent of feeding status [19]. When laser microsurgery was used to ablate ASH, animals failed to respond to 30% and 10% octanol, both on and off food [19]. Combined with our results above, these studies suggest the DOP-3 might act directly in ASH to modulate octanol sensitivity. To determine whether DOP-3 is expressed in ASH (or AWB/ADL), animals expressing a *dop-3::rfp* integrated transgene (*vsIs33*) were crossed to animals carrying integrated transgenes marking each of the octanol-detecting neurons (Figure 6). Surprisingly, DOP-3::RFP expression was not observed in ASH (*osm-10::gfp)*, AWB (*str-1::gfp*) or ADL (*gpa-15::gfp*). Low-level expression was often seen in the ASK sensory neurons that do not detect octanol. Due to their exposed dendritic endings, the head sensory neurons ASH, AWB, ADL, ASJ, ASI and ASK take up lipophilic dyes that mark their cell bodies and projections [39]. Dye-filling experiments confirmed that DOP-3::RFP is not expressed in ASH, AWB or ADL, while weak expression was seen in ASK. DOP-3::RFP expression was also not observed in ASJ or ASI (Figure S1).

### DOP-3 Expression in the ASH Neurons Is Sufficient to Dampen Octanol Sensitivity

**Figure 7:**
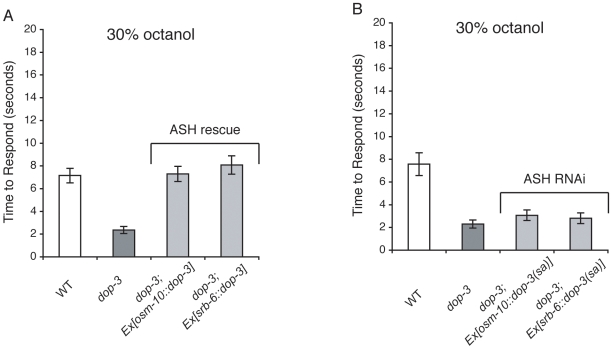
DOP-3 expression in the ASH sensory neurons is sufficient and necessary to modulate sensitivity to dilute octanol. Only the ASH neurons detect dilute octanol [19]. (A) *dop-3(vs106)* animals with DOP-3 expression rescued in ASH are no longer hypersensitive to dilute (30%) octanol (p>0.3 when compared to wild-type animals for both transgenes). The *osm-10* promoter [40] was used to drive DOP-3 expression in the ASH and ASI head neurons. The *srb-6* promoter [9] was used to drive DOP-3 expression in the ASH, ADL and ADF head neurons. (B) RNAi knock-down of *dop-3* in the ASH sensory neurons of otherwise wild-type animals, using the *osm-10* [40] or *srb-6* [9] promoter to co-express *dop-3* sense and antisense (*sa*) sequence, resulted in behavioral sensitivity to dilute (30%) octanol, similar to *dop-3(vs106)* animals (p>0.2 when compared to *dop-3(vs106)* animals for both trangenes). The time to respond is shown. The combined data of ≥3 independent transgenic lines is included for each experiment, n≥60 transgenic animals. Allele used: *dop-3(vs106)*. WT  = the N2 wild-type strain. Error bars represent the standard error of the mean (SEM).

Although we did not observe DOP-3::RFP expression in the octanol detecting neurons, it is possible that the transgene is not expressed in all of the cells that endogenous DOP-3 functions in. It is also possible that the DOP-3::RFP expression levels in some cells are too low to be easily visualized. To determine whether DOP-3 expression in octanol-sensing neurons is sufficient to regulate octanol sensitivity, we used cell-selective promoters to rescue DOP-3 expression in *dop-3* mutant animals. The *osm-10* promoter drives expression strongly in ASH and weakly in the ASI head neurons [40]. The *srb-6* promoter drives expression in the ASH, ADL and, to a lesser extent, ADF head neurons [9]. Both the *osm-10::dop-3* and *srb-6::dop-3* transgenes dampened the hypersensitive response of *dop-3* mutants to 30% octanol, so that the response of transgeneic animals to dilute octanol was similar to wild-type animals (Figure 7A). As the ASH neurons are the only head sensory neurons that both of these promoters are expressed in, we conclude that DOP-3 expression in ASH is sufficient to modulate behavioral sensitivity to dilute (30%) octanol.

### Loss of DOP-3 Function in the ASH Neurons Leads to Octanol Hypersensitivity

To determine whether selective loss of endogenous DOP-3 function in the ASH sensory neurons could also lead to octanol hypersensitivity, we used the cell-specific RNAi approach of Esposito et al. [41] to knock down *dop-3* in ASH. Either the *osm-10* [40] or the *srb-6* [9] promoter was used to co-express a *dop-3* fragment (corresponding to exons 6–9) in both the sense and antisense (*sa*) orientations in the ASH neurons of otherwise wild-type animals. *dop-3* knock-down using either promoter resulted in hypersensitive responses to 30% octanol, similar to *dop-3(vs106)* animals (Figure 7B). This suggests that, although we did not observe DOP-3::RFP transgene expression in ASH, endogenous DOP-3 normally functions in the ASH sensory neurons to dampen sensitivity and behavioral responses to dilute octanol.

## Discussion

In both vertebrates and invertebrates, biogenic amines contribute to multiple forms of behavioral plasticity ranging from learning and memory to sensitization and tolerance in drug addiction [42]–[48]. In *C. elegans*, DA modulates a form of non-associative learning and memory called “tap habituation”; animals lacking DOP-1 receptor function habituate to non-localized mechanical stimulation (“tap” of the culture plate) faster than wild-type animals [35], [49]–[51]. However, we still know very little about the molecular mechanisms that contribute to these diverse forms of behavioral plasticity across species. While the human brain contains over 100 billion neurons, the entire *C. elegans* nervous system consists of just 302 neurons and the physical positions and synaptic connectivity of all of the neurons are known [52]–[54]. This compact, well-characterized nervous system, combined with a sophisticated repertoire of sensory behaviors, makes *C. elegans* an excellent system in which to identify and functionally characterize molecular mechanisms that underlie neuronal signal transduction and regulation.

As *C. elegans* navigate their natural soil environment, they encounter sensory signals of varying strengths. In addition, their behavioral responses to these cues are context and experience dependent [1], [31], [32]. Notably, the feeding status of an animal can rapidly and reversibly affect its behavioral sensitivity to aversive stimuli [19]. Such plasticity may allow animals that are well fed to be very sensitive to noxious stimuli to avoid potentially harmful environments, while starving animals may not have the luxury of being so discriminatory; starved animals might take greater risks as they search for food in their environment [19]. Importantly, *C. elegans* utilize biogenic amines to modulate aversive chemosensory responses as well as olfactory adaptation to attractive chemical cues [10], [13], [18]–[20], [55], [56].

*C. elegans* hermaphrodites have eight dopaminergic neurons that are believed to release DA in response to mechanical stimulation, such as from moving through a bacterial food source [21], [57]. As in mammals [58]–[60], DA can act at a distance (extrasynaptically) in *C. elegans* [21], [25], [35]. Thus, although the synaptic connectivity of the *C. elegans* nervous system is known, it does not allow for direct prediction of the site of DA function for a given behavior. Therefore, understanding the contribution of individual receptors and where they are functioning should prove useful in understanding how DA modulates specific behaviors.

While DOP-3 has been shown to affect *C. elegans* locomotion behaviors [21], [25], [61], a specific role in sensory signaling was not previously known. We show here that DOP-3 is required for the ability of both food, which stimulates endogenous DA release [21], and exogenous DA to rescue the octanol avoidance defect of *rgs-3* mutant animals. In addition, animals lacking DOP-3 function are hypersensitive to dilute octanol, further suggesting that DOP-3 mediates the inhibitory effects of endogenous DA on chemosensory signaling.

As the receptors for biogenic amines sometimes function in cells besides ASH to modulate ASH-mediated behavioral responses [18], [20], we sought to characterize DOP-3 expression. We did not observe DOP-3 expression in any of the three octanol-detecting neurons (ASH, AWB or ADL) and previous analysis did not identify DOP-3 expression in the command interneurons that are the downstream synaptic targets of the sensory neurons [25]. Since *cat-2* and *dop-3* mutants are hypersensitive to dilute (30%) octanol, theoretically DA could normally dampen chemosensory response in wild-type animals by acting on either the chemosensory neurons or the interneurons. However, because DA rescues the responses of *rgs-3* mutant animals, which have behavioral defects due to enhanced signaling in the chemosensory neurons but decreased glutamatergic signaling at the chemosensory/interneuron synapse [13], DA likely dampens signaling in chemosensory neurons. Accordingly, using two different promoters to drive transgenic expression of DOP-3 in ASH, we found that DOP-3 function in ASH is sufficient to modulate octanol sensitivity. In addition, RNAi knock-down of *dop-3* in ASH (using the same two promoters) results in octanol hypersensitivity similar to *dop-3(vs106)* loss-of-function animals. Taken together, our data suggest that endogenous DOP-3 likely acts to dampen chemosensory signaling in the ASH sensory neurons that directly detect octanol.

This is consistent with DOP-3 belonging to the D2-like receptor class, which generally leads to decreased adenylate cyclase activity via G_i_α/G_o_α [30]. Although the *C. elegans* ASH sensory neurons appear to use polyunsaturated fatty acids (PUFAs) instead of cAMP as second messengers [62], mammalian D2-like receptors (D2, D3 and D4) have been shown to affect phospholipase activity and PUFA signaling in Gα-independent ways [30]. For example, while D2 and D4 potentiate arachadonic acid signaling [63]–[66], D3 signaling may be inhibitory [67]. In addition, D2 stimulates phospholipase D cleavage of phosphatidylcholine to increase choline and phosphatidic acid levels [30], [68], [69]. Thus, DOP-3 activity may also regulate chemosensory second messenger signaling in the ASH neurons of *C. elegans*.

Biogenic amines can interact in complex ways to ultimately regulate cellular and whole animal responses. For example, although DA, TA and OA all seem to counteract 5-HT in the regulation of *C. elegans* response to octanol [13], [18], DA can also counteract OA signaling in the cholinergic SIA interneurons to regulate food response [70]. In addition, although both 5-HT and DA decrease the rate of *C. elegans* locomotion, they have antagonistic effects on egg laying [21], [24], [27], [28], [31], [32]. Further complicating matters, DA signaling through DOP-1 and DOP-3 actually has opposing effects on locomotion [25]. Signaling in the human brain is, no doubt, also a fine balance between stimulatory and inhibitory pathways. Use of model organisms such as *C. elegans* should continue to advance our understanding of biogenic amine function and the interaction between modulatory pathways that regulate signaling to ultimately control animal behavior.

## Materials and Methods

### Strains

Strains were maintained under standard conditions on NGM agar plates seeded with OP50 *E. coli* bacteria [71]. Strains used in this study include: N2 Bristol wild-type, LX242 *rgs-3(vs19)*, LX636 *dop-1(vs101)*, LX702 *dop-2(vs105)*, LX703 *dop-3(vs106)*, FG58 *dop-4(tm1392)*, LX705 *dop-1(vs100)dop-3(vs106)*, FG25 *rgs-3(vs19);dop-1(vs101)*, FG27 *rgs-3(vs19);dop-2(vs105)*, FG29 *rgs-3(vs19);dop-3(vs106)*, FG81 *rgs-3(vs19);dop-4(tm1392)*, FG86 *rgs-3(vs19);dop-1(vs100)dop-3(vs106)*, CB1112 *cat-2(e1112)*, HA1739 *rgs-3(vs19);cat-2(e1112)*, FG83 *vsIs33[dop-3::rfp]*, FG94 *vsIs33[dop-3::rfp];rtIs27[osm-10::gfp]*, FG100 *vsIs33[dop-3::rfp];kyIs104[str-1::gfp]*, FG101 *vsIs33[dop-3::rfp];pkIs591[dpy-20(+) + gpa-15::gfp]*, FG157 *dop-3(vs106);udEx7[osm-10::dop-3]*, FG158 *dop-3(vs106);udEx8[osm-10::dop-3]*, FG161 *dop-3(vs106);udEx9[srb-6::dop-3]* and FG162 *dop-3(vs106);udEx10[srb-6::dop-3]*, FG196 *udEx43[osm-10::dop-3(sense + antisense)]*, FG197 *udEx44[osm-10::dop-3(sense + antisense)]*, FG194 *udEx41[srb-6::dop-3(sense + antisense)]*, FG195 *udEx42[srb-6::dop-3(sense + antisense)]*.

### Plasmid Construction

pFG11 *osm-10::dop-3(genomic)*: The *unc-47* promoter was removed from pCL35 *unc-47::dop-3(genomic*) [25] using SphI and BamHI. The ∼900 bp *osm-10* upstream promoter region was isolated from CR142 [72] using the same enzymes and was inserted into the SphI/BamHI sites upstream of the *dop-3* genomic clone in the remaining fragment of pCL35.

pFG12 *srb-6::dop-3(genomic)*: The ∼1.3 kb *srb-6* promoter was first isolated from pHA#355 [12] using PstI and BamHI and inserted into the same sites of Fire vector pPD49.26 to create pFG10. The *srb-6* promoter was then removed from pFG10 using SphI and BamHI to be inserted into the SphI/BamHI sites upstream of the *dop-3* genomic clone (remaining fragment of pCL35) as described above.

### Transgenic Strains

Germline transformations were performed as previously described [73]. For *dop-3* rescue experiments 75 ng/µl of pJM67 *elt-2::gfp* plasmid [74] was used as the co-injection marker, along with 50 ng/µl of either pFG11 *osm-10::dop-3(genomic)* or pFG12 *srb-6::dop-3(genomic)*. For cell-specific RNAi transgenic experiments 25 ng/µl of pJM67 *elt-2::gfp* plasmid [74] was co-injected with 40–50 ng/µl each of PCR fusion product [41] corresponding to *osm-10::dop-3(sense)* and *osm-10::dop-3(antisense)* or *srb-6::dop-3(sense)* and *srb-6::dop-3(antisense)*. Exons 6–9 (∼840 bp) of *dop-3* were amplified from genomic DNA to generate the sense and antisense fragments; no functional protein should be made from this internal region. Primer sequences are available upon request.

### RNAi Experiments

Cell-specific RNAi knock-down experiments were performed as previously described [41], using the above amounts of injected DNA (see Transgenic Strains).

### Behavioral Assays

Well-fed young adult animals were used for analysis, and all behavioral assays were performed on at least two separate days, along with controls. Behavioral assays were performed as previously described [9], [13], [40]. Response to octanol was scored as the amount of time it took an animal to initiate backward locomotion when presented with a hair dipped in octanol. (Assays were stopped at 20 seconds.) For dilute octanol assays, the octanol was diluted by volume in 100% ethanol. Animals were tested 10–20 minutes after transfer to NGM plates lacking bacteria (“off food”) or NGM plates with a thin lawn of OP50 *E. coli* bacteria (“on food”). As dopamine (DA) is unstable in the presence of salt, 6 mM DA plates were made by spreading 60 µl of a freshly made 1 M stock (dissolved in water) on the surface of a 10 ml NGM plate. The DA was allowed to soak into the plate and equilibrate for 5 minutes prior to the assay, as previously described [13]. Dopamine (hydrochloride complex) was purchased from Sigma. All data is presented as ± standard error of the mean (SEM). The Student's t-Test was used for statistical analysis.

### Neuronal Identification

Animals carrying the integrated transgene *vsIs33*, which encodes *dop-3::rfp*, were crossed to animals carrying integrated transgenes marking selected sensory neurons. ASH was marked by *rtIs27* (*osm-10::gfp*), AWB was marked by *kyIs104* (*str-1::gfp*), and ADL and ASK were marked by *pkIs591* (*gpa-15::gfp*). *pkIs591* is also expressed in ASH, which is not visible in the focal plane shown in Figure 6. To label dye-filling sensory neurons, *dop-3::rfp* (*vsIs33*) expressing animals were incubated with the lipophilic dye DiO (Molecular Probes, Invitrogen), as previously described [39]. Images were obtained using a Zeiss Axio Imager Z1 microscope (using a 63x Plan-APO oil objective, epi-fluorescence and DIC optics), high resolution AxioCam MRm digital camera and Zeiss AxioVision software.

## Supporting Information

Figure S1 DOP-3::RFP is not expressed in the sensory neurons that detect octanol. Six head sensory neurons (ASH, AWB, ADL, ASJ, ASI and ASK) take up lipophilic dyes via their exposed sensory endings [39]. Animals expressing DOP-3::RFP from the integrated transgene *vsIs33* were incubated with DiO, shown in green, to mark the cell bodies and projections of these neurons. DOP-3::RFP expression was not seen in ASH, AWB, ADL, ASJ or ASI. Weak DOP-3::RFP expression was often observed in ASK. Scale bar  = 20 µm.
(10.18 MB TIF)
