# Supplementary figures and images for: The C. elegans D2-Like Dopamine Receptor DOP-3 Decreases Behavioral Sensitivity to the Olfactory Stimulus 1-Octanol
Source: PLoS One. 2010 Mar 2;5(3):e9487. doi: 10.1371/journal.pone.0009487 (PMC2830454; doi:10.1371/journal.pone.0009487)

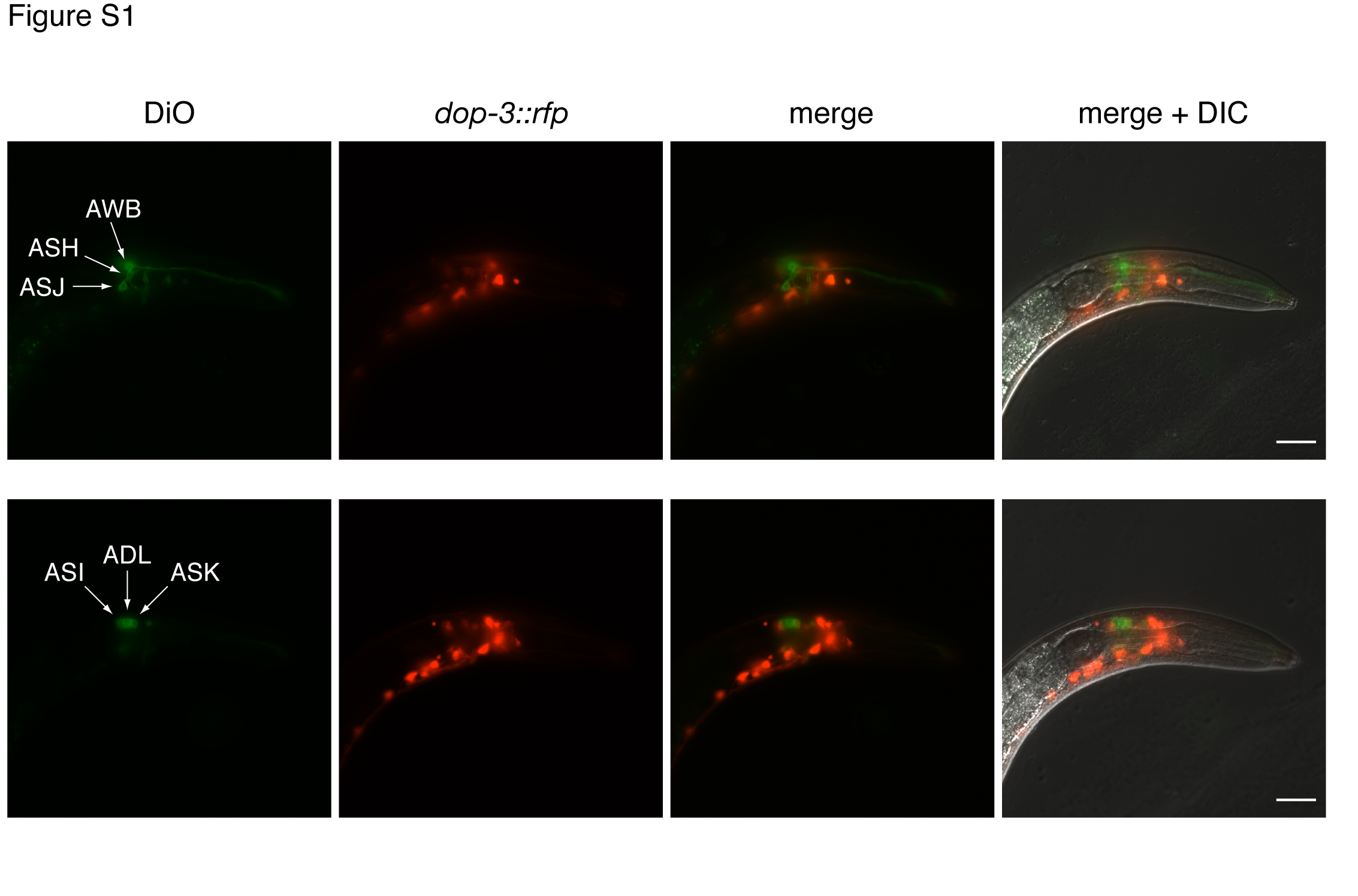

Supplement: Figure S1 — DOP-3::RFP is not expressed in the sensory neurons that detect octanol. Six head sensory neurons (ASH, AWB, ADL, ASJ, ASI and ASK) take up lipophilic dyes via their exposed sensory endings [39]. Animals expressing DOP-3::RFP from the integrated transgene vsIs33 were incubated with DiO, shown in green, to mark the cell bodies and projections of these neurons. DOP-3::RFP expression was not seen in ASH, AWB, ADL, ASJ or ASI. Weak DOP-3::RFP expression was often observed in ASK. Scale bar = 20 µm. (10.18 MB TIF) [file pone.0009487.s001.tif]
